# Hypoxia-Inducible Factor-2α as a Novel Target in Renal Cell Carcinoma

**DOI:** 10.15586/jkcvhl.v8i1.170

**Published:** 2021-04-07

**Authors:** Won Seok W. Choi, Julia Boland, Jianqing Lin

**Affiliations:** George Washington University Hospital, School of Medicine and Health Sciences, George Washington University, Washington, DC, USA

**Keywords:** clear cell renal cell carcinoma, HIF-2α inhibitors, hypoxia-inducible factor, pseudohypoxia, von Hippel–Lindau)

## Abstract

Hypoxia-inducible factor (HIF), an important mediator of hypoxia response, is implicated in tumorigenesis in the setting of pseudohypoxia, such as in the inactivation of von Hippel–Lindau tumor suppressor protein (pVHL), leading to development and progression of clear cell renal cell carcinoma (ccRCC). Targeting downstream molecules in HIF pathway, such as vascular endothelial growth factor (VEGF), has led to improvement in clinical outcome for patients with advanced ccRCC, but such therapy thus far has been limited by eventual resistance and treatment failure. Following the discovery of HIF-2α playing a key role in ccRCC carcinogenesis, inhibitors targeting HIF-2α have been developed and have demonstrated encouraging efficacy and safety profile in clinical trials. This review discusses HIF-2α as a promising therapeutic target for ccRCC.

## INTRODUCTION

Renal cell carcinoma (RCC) is the most common type of kidney cancer, accounting for 90% of all kidney cancers (1). Clear cell renal cell carcinoma (ccRCC) is the most common subtype of RCC, accounting for approximately 75% of patients (2). The earliest genetic event in most ccRCC is a loss-of-function mutation in *von Hippel–Lindau* (*VHL*) tumor suppressor gene (3). Loss of both *VHL* gene alleles through deletion, mutation, or other mechanisms results in loss of function of von Hippel–Lindau tumor suppressor protein (pVHL). This leads to the upregulation of hypoxia-inducible factor (HIF) and resultant overexpression of hypoxia-inducible genes, such as vascular endothelial growth factor (VEGF), platelet-derived growth factor-β (PDGF-β), and transforming growth factor-α (TGF-α) (VHL-HIF-VEGF axis), that are involved in tumorigenesis and progression of ccRCC (4).

## BODY

### Regulation of HIF-1α and HIF-2α

Hypoxia-inducible factor is a transcription factor composed of oxygen-sensitive alpha subunit (HIF-α) and constitutively expressed beta subunit (HIF-b), which is also known as aryl hydrocarbon receptor nuclear translocator (ARNT) (5). There are three isoforms of the alpha subunit: HIF-1α, HIF-2α, and HIF-3α. Of these three, the first two are the best studied isoforms. HIF-1α and HIF-2α each has two transactivation domains (TAD), one at the NH2 terminal (N-TAD) and another at the COOH terminal (C-TAD). C-TAD interacts with p300/cAMP-response element binding protein (CREB)-binding protein (CBP) to modulate transcription in hypoxic conditions. N-TAD stabilizes HIF-α against degradation (5). Oxygen-dependent degradation domain (ODDD) is positioned within N-TAD and contains specific proline residues (4). Variability between HIF-1α and HIF-2α is observed mostly within N-TAD (6), whereas C-TADs between HIF-1α and HIF-2α isoforms have 67% similarity and promote the expression of their common target genes (7).

HIF-1α and HIF-2α are finely regulated in response to different oxygen states. In normoxic conditions, both HIF-1α and HIF-2α are degraded via the pVHL/E3-ubiquitin ligase pathway (5). The proline residues in ODDD are hydroxylated by proline hydroxylases (PHDs), which are then bound by pVHL/E3-ubiquitin ligase complex. Ubiquitin ligase adds poly-ubiquitin to HIF-α and marks it for proteasomal degradation (5).

In hypoxic conditions, PHD loses its activity and prevents VHL binding, leading to stabilization and accumulation of HIF-α (4). In pseudo-hypoxic conditions, pVHL is inactivated due to loss-of-function mutation, which also leads to accumulation of HIF-α (8). HIF-α then translocates into the nucleus, where it heterodimerizes with HIF-b and recruits p300/CBP co-activators to form an active HIF transcription complex (4). This complex binds to hypoxia response elements (HREs) on hypoxia-sensitive genes and activates transcription (4). Activation of HIF transcription leads to upregulation of hypoxia-inducible genes such as VEGF (2). These genes are involved in processes such as cellular metabolism, glucose transport, angiogenesis, erythropoiesis, iron metabolism, pH regulation, apoptosis, and cell proliferation, thus promoting and leading to the progression of ccRCC (5).

**Figure 1:**
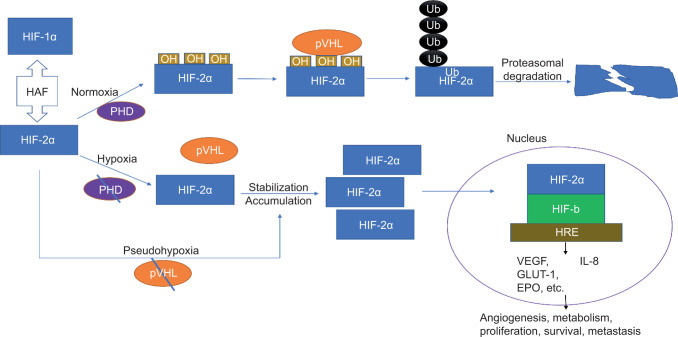
HIF-2α pathway.

Switch from HIF-1α- to HIF-2α-dependent transcription is the result of hypoxia-associated factor (HAF) activity (6). HAF is a HIF-1α-specific E3 ligase that causes HIF-1α ubiquitination and proteasomal degradation. HAF also increases transcriptional activity of HIF-2α, independent of its E3 ligase activity. HAF level decreases in acute hypoxia, allowing HIF-1α to predominate, but HAF level increases in chronic hypoxia, which is mediated predominantly by HIF-2α (6). The HIF pathway focusing here on HIF-2α is illustrated in Figure 1.

There are other mechanisms for stabilization of HIF that involve inhibition of PHDs. Mutations in *succinate dehydrogenase* (*SDH*) and *fumarate hydratase* (*FH*) genes, which are Kreb’s cycle enzymes and act as tumor suppressor genes, lead to the accumulation of succinate and fumarate, respectively, in the cytosol. Succinate and fumarate inhibit PHDs and prevent hydroxylation of HIF-1α and HIF-2α, thus leading to their stabilization. This also creates a pseudohypoxic state in which hypoxia response pathways are aberrantly activated in spite of normoxic conditions (6). Germline FH mutations are implicated in hereditary leiomyomatosis and renal cell carcinoma (HLRCC) syndrome. Additionally, similar mechanisms of HIF stabilization are seen with mutations in *succinate dehydrogenase complex assembly factor 2* (*SDHAF2*), *hypoxia-inducible factor prolyl hydroxylase 1/2/Egl-9 homolog½* (*EGLN1/2*), and *endothelial PAS domain-containing protein 1* (*EPAS1*) genes (9).

### Role of HIF-2α in ccRCC carcinogenesis

The constitutive activation of the VHL-HIF-VEGF axis is the key mechanism of ccRCC carcinogenesis (2). Inactivation of *VHL* gene, which is almost always the first step in ccRCC carcinogenesis (10), leads to stabilization and transcription of HIF, creating a pseudohypoxic state. Transcription of HIF, and HIF-2α in particular, leads to overexpression of hypoxia-inducible genes, including VEGF, PDGF-β, TGF-a, c-Met, cyclin D1, and stromal cell-derived factor 1 (SDF1) and its receptor CXC chemokine receptor 4 (CXCR4) (10), which are involved in tumor angiogenesis, cell proliferation, survival, metabolism, invasion, metastasis, therapy resistance, inflammation, and immunity (4).

While HIF-1α and HIF-2α share a similar protein structure, they have several significant differences in hypoxia response and gene regulation. HIF-1α predominantly mediates response to acute hypoxia, whereas HIF-2α mediates response to chronic hypoxia (6, 11). Likewise, the two isoforms of HIF-α have different gene targets and regulatory functions. For example, while both carbonic anhydrase IX (CAIX) and glucose transporter 1(GLUT-1) are overexpressed in many forms of cancer, including RCC, CAIX was found to be negatively regulated by HIF-1α whereas GLUT-1 was found to be a specific HIF-2α target in VHL-defective RCC (12). Important differences between HIF-1α and HIF-2α are highlighted in Table 1.

**Table 1:** Differences between HIF-1α and HIF-2α.

	HIF-1α	HIF-2α
Hypoxia response	Acute hypoxia	Chronic hypoxia
Targets	CAIX, PDK, BNip3, Mxi-1, VEGF, EPO	GLUT-1, Cyclin D1, TGF-α, VEGF, EPO
Oxidative phosphorylation	–	+
Glycolysis	+	–
Carcinogenesis	–	+
Upregulation of c-Myc, IL-8	–	+

– : Negative or no impact; +: Promote.

HIF-1α is primarily involved in glucose metabolism, upregulating glycolytic enzymes while limiting pyruvate uptake by mitochondria and downregulating electron transport chain (ETC) activity (13). Most epithelial cancer cells rely on HIF-1α transcriptional products to mediate tumor metabolism such as Warburg effect, which leads to reprogramming of tumor cells from mitochondrial respiration via oxidative phosphorylation to glycolysis. Pyruvate dehydrogenase (PDH), a key enzyme that links glycolysis to TCA cycle, is negatively regulated by pyruvate dehydrogenase kinase (PDK). HIF-1α leads to high levels of PDK and thus low levels of PDH, leading to more glycolysis and anaerobic metabolism (14).

In contrast, HIF-2α is uniquely involved in tumor growth and cell cycle progression through interaction with proto-oncogene c-Myc (14). Expression of HIF-2α upregulates proteins involved in cell proliferation (cyclin D1), cell growth (TGF-α), and angiogenesis (VEGF) (14). Expression of HIF-2α can lead to a more oxidative tumor phenotype, which in turn promotes a more aggressive tumor with heightened treatment resistance (14). For instance, HIF-2α leads to lower PDK and higher PDH, leading to more oxidative phosphorylation, rather than glycolysis (14).

In the context of ccRCC associated with VHL’s loss of function, it appears that HIF-2α plays a key role in carcinogenesis. Forced expression of HIF-2α, not HIF-1α, antagonizes pVHL’s tumor-suppressor activity in nude mice models (14). Studies have shown that many ccRCC cell lines have sustained homozygous deletions that specifically inactivate HIF-1α and solely produce HIF-2α (15), which suggests that HIF-2α, not HIF-1α, is essential for carcinogenesis of ccRCC. It was also observed that ccRCC tumors expressing only HIF-2α were larger and more resistant to replicative stress compared to those that express both HIF-1α and HIF-2α (14).

While HIF-2α functions as a driver of tumorigenesis, HIF-1α appears to act as a tumor suppressor (16). HIF-1α positively regulates Bcl2 interacting protein (BNip3), a member of the Bcl-2 family of pro-apoptotic proteins having anti-tumorigenic properties (12). Another important mediator in tumorigenesis is interleukin 8 (IL-8), which promotes angiogenesis and is associated with treatment resistance (17). HIF-1α decreases expression of IL-8 via downregulation of nuclear factor erythroid 2-related factor 2 (Nrf2) and c-Myc and upregulation of max-interacting protein 1 (Mxi-1), a c-Myc antagonist; on the other hand, HIF-2α upregulates IL-8 expression by increasing Sp-1 and c-Myc activity (6). See Table 1.

### VHL disease and HIF

VHL disease is a hereditary disease transmitted in an autosomal dominant fashion that is caused by mutation of *VHL* gene and leads to the formation of tumors in multiple organs, including central nervous system (CNS) hemangioblastomas, RCCs, pheochromocytomas, and pancreatic neuroendocrine tumors (PNETs) (18). CNS hemangioblastomas are among the most common tumors found in patients with VHL disease, most commonly affecting the cerebellum, brainstem, spinal cord, and retina (18). As discussed earlier, HIF is upregulated by loss-of-function mutation in *VHL* gene and in turn pVHL, resulting in increased expression of numerous genes implicated in tumorigenesis, including VEGF. This pVHL-HIF-VEGF pathway is responsible for tumorigenesis in not only RCC but also CNS hemangioblastomas and PNETs in VHL disease (18). Thus far, VEGF inhibitors have been shown to demonstrate limited efficacy in treatment of CNS hemangioblastomas. Pazopanib, a multikinase inhibitor, was studied in a phase-II trial of patients with VHL-associated lesions (NCT01436227) (19). While responses were seen in 52% of RCCs and 53% of PNETs, only 4% of patients with CNS hemangioblastomas showed response (19). Sunitinib, another multikinase inhibitor, was studied in patients with VHL disease, and led to partial response in 33% of RCCs but none in hemangioblastomas (20). Theoretically, targeting the upstream molecule such as HIF-2α will be more effective than VEGF receptors inhibition in treating VHL disease. There is growing optimism that novel HIF-2α inhibitors will improve outcomes in patients with VHL-associated lesions, including non-renal lesions such as hemangioblastomas. The efficacy and safety data and potential role of HIF-2a inhibitors in VHL disease is discussed below in detail.

### Treatment resistance and development of HIF-2a inhibitor in ccRCC

After the discovery of VHL-HIF-VEGF axis and its role in ccRCC carcinogenesis, multiple agents have been developed to inhibit specific molecular targets in the HIF pathway. For instance, VEGF inhibitors, such as sunitinib and cabozantinib, have been developed to target the gene product VEGF, which is downstream in the HIF pathway (9), and they remain the mainstay of current ccRCC treatment. Other drugs (everolimus and temsirolimus) targeting phosphoinositide 3-kinase/protein kinase B/mechanistic target of rapamycin (PI3K/AKT/mTOR) pathway have been developed and gained regulatory approval for the treatment of advanced RCC (17).

However, these treatment strategies are limited by eventual development of treatment resistance (9). Inhibition of one part of HIF pathway may trigger compensatory mechanisms that overproduce other alternative proangiogenic factors, leading to drug resistance. For example, in ccRCC, inhibition of one angiogenic factor (e.g., VEGF) may lead to an increase in the expression of other angiogenic factors (e.g., interleukin 6 [IL-6], IL-8, monocyte chemoattractant protein 1 [MCP-1], and basic fibroblast growth factor [bFGF]), leading to upregulation of angiogenesis, rather than its downregulation (6). Similarly, studies involving HIF-1α inhibitors, which were developed in a hope of improved control of advanced ccRCC by targeting the HIF pathway more proximally, showed that inhibition of HIF-1α may lead to compensatory upregulation of HIF-2α, which can inadvertently lead to more neo-angiogenesis and tumor progression (4, 6). One strategy to overcome treatment resistance is to combine therapeutic agents with different molecular targets, such as combining different tyrosine kinase inhibitors (TKIs) with each other or with mTOR inhibitors. Other strategies involve combination of TKIs with immune checkpoint inhibitors such as programmed cell death protein 1 (PD-1) inhibitors, programmed death ligand 1 (PD-L1) inhibitors, and cytotoxic T-lymphocyte-associated protein 4 (CTLA-4) inhibitors (17).

In addition to investigating different combination regimens to circumvent treatment resistance, recent studies have focused on developing new therapeutic agents with novel molecular targets. Given the evidence that HIF-2α is the main driver of tumorigenesis in ccRCC (14, 21), as well as the theoretical advantage of targeting the VHL-HIF-VEGF pathway more upstream, there has been growing interest in the therapeutic benefit of HIF-2α inhibition. HIF-2α was initially thought of as an undruggable target, but the structure-activity relationship study of small molecules designed to inhibit HIF-2α-ARNT heterodimerization led to the development of inhibitors that target HIF-2α (22). PT2399 and PT2385, the first HIF-2α inhibitors being developed, inhibit dimerization of HIF-2α and ARNT1 and HIF-2α-dependent transcription (23). PT2399 was shown to dissociate specifically HIF-2α *in vitro*, resulting in reduced expression of HIF-2α target genes but not HIF-1α target genes in human ccRCC cell lines. This was reviewed by Chen et al. (23). There was reported resistance to PT2399 already. It was shown that in resistant tumors, even though there was evidence of HIF-2α disassembly, most of its target genes were left unaffected. Experiments in RCC cell lines suggested that tumor protein P53 (TP53) mutations may confer resistance to HIF-2α inhibitors, but the extent to which these mutations result in resistance remains to be determined (24).

PT2385, a close analog of PT2399, was studied in a phase-I clinical trial that evaluated maximum tolerated dose in patients with locally advanced or metastatic ccRCC and Eastern Cooperative Oncology Group (ECOG) performance score of 0 or 1 who suffered disease progression despite previous treatment with a VEGF inhibitor (18). PT2385 did not cause dose-limiting toxicity at any dose level tested and was shown to have a favorable safety profile. Most adverse events were of grade 1 or grade 2, which included anemia (45%), peripheral edema (39%), and fatigue (37%). The most common adverse events that were of grade 3 or grade 4 included anemia (10%), hypoxia (10%), and lymphopenia (8%). Adverse events did not lead to discontinuation in treatment or death in any of the patients. Unlike VEGF inhibitors, PT2385 did not lead to hypertension, thromboembolic events, or other cardiotoxicity. Likewise, PT2385 showed promising efficacy in patients who have already received multiple treatments for ccRCC, with complete response (CR) of 2%, partial response (PR) of 12%, and stable disease (SD) in 52% of patients (18). Disease control rate (CR + PR + SD) was 66%, and 42% of patients had SD, PR, or CR for at least 4 months. At a median follow-up of 17.5 months, 25% of patients had a progression-free survival (PFS) of >14 months. However, PT2385 was shown to have variable pharmacokinetics (25), with greater exposure correlated with longer PFS (21).

PT2977 (also known as MK-6482) is a second-generation HIF-2a inhibitor that has improved pharmacokinetic profile, selectivity, and potency than PT2385 (22). Phase-I/II trial evaluated the safety and efficacy of MK-6482 in 55 patients with previously treated advanced ccRCC (23). Most patients (67%) had received prior treatment with VEGF inhibitor and immune checkpoint inhibitor. Overall response rate (ORR) was 24%. Thirty-one patients (56%) had SD, and the disease control rate was 80%. The median duration of response (DOR) was not reached at the time report. The median PFS was 11 months. Anemia (26%) and hypoxia (15%) were the most common grade-3 adverse reactions. No grade-4/5 drug-related adverse events were observed; 4% of patients discontinued treatment due to drug toxicity. It was concluded that MK-6482 was well tolerated with a favorable safety profile and demonstrated promising single-agent activity in heavily pretreated patients with ccRCC across International Metastatic RCC Database Consortium (IMDC) risk groups (23). A phase-III trial in a similar population is currently ongoing (Table 2).

**Table 2:** Selected clinical trials using HIF-2 inhibitors in clear cell renal cell carcinoma (ccRCC) and other tumors.

Title of the trial	Cancer type	Phase	Primary endpoint	Trial identifier
A phase-I, dose-escalation trial of PT2385 tablets in patients with advanced ccRCC	ccRCC	I	MTD	NCT02293980
A trial of PT2977 tablets in patients with advanced solid tumors	Solid tumors, ccRCC, GBM	I	MTD	NCT02974738
PT2385 for the treatment of VHL disease-associated ccRCC	ccRCC	II	ORR	NCT03108066
A phase-II study of MK-6482 (PT2977) for the treatment of VHL disease-associated RCC (MK6482-004)	RCC	II	ORR	NCT03401788
A study of MK-6482 in combination with lenvatinib versus cabozantinib for treatment of RCC (MK-6482-011)	ccRCC	III	PFS, OS	NCT04586231
A study of MK-6482 versus everolimus in patients with advanced RCC (MK-6482-005)	ccRCC	III	PFS, OS	NCT04195750

Source: clinicaltrials.gov. Accessed October 30, 2020. MTD: maximum tolerated dose; ORR: overall response rate; PFS: progression-free survival; OS: overall survival.

A phase-II trial evaluating MK-6482 showed promising clinical activity in treatment-naïve patients with VHL-associated non-metastatic ccRCC (24). Most common non-renal lesions were CNS hemangioblastomas (80.3%) and pancreatic lesions (50.8%). ORR was 27.9% (17/61), and efficacy was durable in both RCC and non-renal lesions. Complete responses were observed in 6.6% (4/61) of pancreatic lesions and 11.6% (5/43) of CNS hemangioblastomas (24). The median DOR and median PFS were not reached. Most adverse events were of grade-1 or grade-2, and the most common adverse events included anemia (83.6%), fatigue (49.2%), and dizziness (21.3%). Grade-3 adverse events occurred in 9.8% of patients. There were no grade-4 or grade-5 events. Based on this data, the US Food and Drug Administration (FDA) granted breakthrough therapy designation to MK-6482 for the treatment of patients with VHL disease-associated RCC with non-metastatic RCC tumors of less than 3 cm in size, unless immediate surgery is required. The FDA also granted orphan drug designation to MK-6482 for VHL disease.

Selected clinical trials studying the HIF-2a inhibitors are summarized in Table 2.

## Future Perspective

HIF-2α is now considered as a novel druggable target for the treatment of ccRCC. Given the favorable safety profile and efficacy of HIF-2α inhibitors, the clinical trials combining HIF-2α inhibitors with TKI, immune checkpoint inhibitors, or other antiangiogenic agents are on the way, and are promising. The future research needs to focus on better understanding of mechanisms of resistance to HIF-2α inhibitors. The regulation of tumor microenvironment and its associated biomarker research are keys for patient selection and success of treatment.

## Conclusion

HIF is the key mediator of oxygen hemostasis, which can be aberrantly activated in pseudohypoxic conditions such as in pVHL-defective ccRCC. HIF-2α, not HIF-1α, is the main driver of carcinogenesis in ccRCC. HIF-2α as a novel target for the treatment of ccRCC is established, and inhibitors to this pathway are being developed actively. Instead of targeting endothelial cell VEGF pathway with TKIs, HIF-2α inhibitors target more proximally and directly the tumor cell HIF pathway. Early-phase clinical trials involving HIF-2α inhibitors have already shown promising efficacy in the treatment of advanced ccRCC.
